# *Phonological Iconicity* Electrifies: An ERP Study on Affective Sound-to-Meaning Correspondences in German

**DOI:** 10.3389/fpsyg.2016.01200

**Published:** 2016-08-18

**Authors:** Susann Ullrich, Sonja A. Kotz, David S. Schmidtke, Arash Aryani, Markus Conrad

**Affiliations:** ^1^Languages of Emotion Research Cluster, Freie Universität BerlinBerlin, Germany; ^2^Experimental and Neurocognitive Psychology, Freie Universität BerlinBerlin, Germany; ^3^Department of Neuropsychology and Psychopharmacology, Maastricht UniversityMaastricht, Netherlands; ^4^Department of Neuropsychology, Max Planck Institute for Human Cognitive and Brain SciencesLeipzig, Germany; ^5^Department of Cognitive, Social, and Organizational Psychology, University of La LagunaTenerife, Spain

**Keywords:** sublexical, lexical, affect, language, EEG, ERPs, *phonological iconicity*, sound-to-meaning correspondences

## Abstract

While linguistic theory posits an arbitrary relation between signifiers and the signified (de Saussure, 1916), our analysis of a large-scale German database containing affective ratings of words revealed that certain phoneme clusters occur more often in words denoting concepts with negative and arousing meaning. Here, we investigate how such phoneme clusters that potentially serve as sublexical markers of affect can influence language processing. We registered the EEG signal during a lexical decision task with a novel manipulation of the words' putative *sublexical affective potential*: the means of valence and arousal values for single phoneme clusters, each computed as a function of respective values of words from the database these phoneme clusters occur in. Our experimental manipulations also investigate potential contributions of formal salience to the *sublexical affective potential*: Typically, negative high-arousing phonological segments—based on our calculations—tend to be less frequent and more structurally complex than neutral ones. We thus constructed two experimental sets, one involving this natural confound, while controlling for it in the other. A negative high-arousing *sublexical affective potential* in the strictly controlled stimulus set yielded an early posterior negativity (EPN), in similar ways as an independent manipulation of *lexical affective content* did. When other potentially salient formal features at the sublexical level were not controlled for, the effect of the *sublexical affective potential* was strengthened and prolonged (250–650 ms), presumably because formal salience helps making specific phoneme clusters efficient sublexical markers of negative high-arousing affective meaning. These neurophysiological data support the assumption that the organization of a language's vocabulary involves systematic sound-to-meaning correspondences at the phonemic level that influence the way we process language.

## Introduction

Most people would probably agree that not all words sound “neutral.” But is it just personal taste or idiosyncratic individual experience that some words sound nicer and others rather harsh to us? Or do, on the contrary, sublexical phonological patterns possess systematic affective connotations? And if so, might these relate systematically to the meaning of words? A potential associative or even physical resemblance between sound and meaning of a word is called *phonological iconicity* in terms of Peirce's typology of semiotic elements (Peirce, 1931; see also Perniss et al., 2010; Aryani et al., 2013; Schmidtke et al., 2014a), challenging the conventional linguistic view that the relationship between the signifier and the signified be arbitrary (de Saussure, 1916). Note that our use of the term “sound” in this paper refers exclusively to phonological constituents of words themselves, not to speaker related issues such as prosody or the speaker's identity or affective state (for research on the latter ones see, for example, Belin et al., 2011; Hellbernd and Sammler, 2016). This conforms with the traditional literature on *sound symbolism*, which also posits that specific speech-sounds—phonemes—words are made of, may carry specific meaning (Jakobson, 1937; Allott, 1995).

Internal relations between phonological aspects and semantic meaning of words show most directly and prominently in onomatopoetic expressions (that typically describe acoustic phenomena by mimicking them): e.g., bears growl, snakes fizz, babies babble, or water splashes, sprinkles, squirts, drops, or drizzles. On a more abstract level, e.g., phonaesthemes involve the correspondence of specific sublexical patterns (typically word initial phoneme clusters) to specific semantic word fields (Firth, 1930). For instance, many English words related to vision and light start with “gl-”: glance, glitter, gloom, glisten, glare, or gloss—while many words related to the nose start with “sn-”: snore, sniff, snort, snuff, snoop, or sneeze (Wallis, 1699; Bloomfield, 1933). Although the reasons for the evolution of phonaesthemes remain somewhat opaque, Bergen (2004) could show in priming experiments that these subtle statistical associations influence language processing. Other systematic sound-to-meaning correspondences have also been found to support word learning (Nygaard et al., 2009; Lockwood et al., 2016).

That the sound of a word and its signified semantic concept may, in general, share a common quality has already been discussed by Socrates in Plato's Cratylus (Plato, 1892). Throughout the last century, a number of empirical psychological studies have investigated how potential correspondences between sublexical language sounds and attributes of meaning influence human perception of, e.g., size, shape, lightness, pleasantness, or excitement. For instance, back vowels (a, o) are perceived as bigger, heavier, or darker than front vowels (i, e), as has been shown, for example, by Sapir (1929) who asked people to connect pseudowords such as MAL and MIL with either a large or a small object. Other researchers replicated and refined these findings on vowels and extended them to consonants, showing, for example, that people perceive front consonants as smaller and more pleasant than back consonants, or voiced consonants as darker and larger than unvoiced consonants (Newman, 1933; Folkins and Lenrow, 1966). In general, such phenomena subsumed under the terms *sound symbolism* or *phonological iconicity* (for reviews see Perniss et al., 2010; Perniss and Vigliocco, 2014; Schmidtke et al., 2014a; Dingemanse et al., 2015) involve the view that that the sound of a word and the signified concept share a common quality (see already von Humboldt, 1836, or Plato, 1892). As a potential cause, it has been proposed that language may have phylogenetically evolved from the imitation of natural sounds (Darwin, 1871; Plato, 1892). Cross-language replications of, e.g., the kiki-bouba phenomenon—people, including toddlers, consistently match pseudowords such as *kiki* or *takete* preferentially to spiky shapes, vs. *bouba* or *baluma* to rounded shapes (Köhler, 1929; Werner, 1934, 1957; Davis, 1961; Maurer et al., 2006; also see Westbury, 2005)—suggest *phonological iconicity* to be a common feature of language in general, spurring theories about the biological origin of language (Ramachandran and Hubbard, 2001).

As communication of affect could be seen as a primordial feature of human communication (Jackendoff, 2002), *phonological iconicity* may well extend to affective meaning communicated through language—potentially since its very origins (see Darwin, 1871; Morton, 1977; Kita, 2008; Perniss and Vigliocco, 2014). The basic dimensions of affective meaning in the most influential emotion models (Wundt, 1896; Russell, 1978, 1980, 2003; Watson and Tellegen, 1985; Bradley et al., 1992) are those of valence and arousal, accounting also for a major amount of variance of semantic meaning according to semantic differential techniques (Osgood and Suci, 1955). Interestingly, analyzing the phonological content of 1000 English words rated for valence and arousal, Heise (1966) found that certain phonemes occur significantly more often in words of a specific affective meaning (see also Whissell, 1999, Whissell, 2000). Conrad et al. (in preparation) recently applied this approach to a large-scale database of over 6000 German words rated for valence and arousal (see also Aryani et al., 2015). Their analyses reveal systematic sound-to-meaning correspondences concerning the use of certain phonemes or phoneme clusters in words of specific valence and arousal ranges—in particular representing a combination of high arousal and negative valence that might be summarized as denoting potential threat. To quantify these patterns, they computed *sublexical affective values* (SAVs) for single sub-syllabic phoneme clusters—representing syllabic onsets, nuclei, and codas—by averaging valence and arousal values of all words these units are part of in the database. The choice of these subsyllabic phonological segments instead of single phonemes is motivated by linguistic theories of syllable segmentation (Davis, 1982; Hall, 1992; Wiese, 1996). Accordingly, both experimental (Nuerk et al., 2000; Brand et al., 2007) and simulation studies (Jacobs et al., 1998) of language processing support the importance of those segments as perceptual units encoding phonology in terms of syllabic onsets, nuclei and codas. Within the German database, SAVs for a number of such phonological segments show significant deviations from neutral global means (Conrad et al., in preparation), suggesting an intrinsic affective potential of specific language sounds, which might accordingly serve as sublexical markers of affect, in particular concerning threat. Following this rationale, the average of SAVs for all phonological segments in a word—henceforth called *sublexical affective potential—*might predict the affective appeal of the whole phonological word form at a sublexical level. Indeed, Conrad et al. (in preparation) reveal significant correlations of this *sublexical affective potential* with lexical valence and arousal ratings across the entire respective word database. These findings interestingly point toward *phonological iconicity* with regard to affective content as a systematic feature determining the organization of language (see also Aryani et al., 2015).

### The present study

In this study, we address the question of whether these numerical measures of SAVs—derived from a large-scale normative database for the German language, reflecting systematic sound-to-meaning correspondences within this database—possess any psychological reality concerning the perception of language. In particular, we ask whether these sound-to-meaning correspondences or the underlying affective *phonological iconicity* of the German language would have any neuroscientific correlates during a standard lexical decision task using EEG measurements. If anything like sublexical markers of affective content, in particular threat, exist, those phonological segments typically occurring in words of high arousal and negative content should leave an impact on brain activity strong enough to be traceable with neuroscientific methods during the time course of language perception.

Furthermore, our study focuses on the potential role of formal salience for processes related to *phonological iconicity*. Concerning sublexical phonological units presumably encoding—according to the analyses of our database—negative high-arousing content, we consistently found structurally rather complex phonological segments (i.e., more than one consonant in a syllabic onset or coda) and phonological segments of low frequency of occurrence to appear preferentially in words of negative and high-arousing meaning. As high arousal is thought of as an early alert indicator attracting attention to potentially relevant stimuli (see Recio et al., 2014, for ERP effects disentangling valence and arousal effects during visual word recognition), it seems intuitive that formal salience could be crucial for making a sublexical unit a most efficient “sign of threat” at the conceptual level.

Event-related potentials (ERPs) obtained via EEG measurement with its high temporal resolution are most suitable to study if, when, and how such phenomena influence cognitive processes. A number of psycholinguistic studies have already investigated effects of lexical affective content during visual word recognition using ERPs. Two main ERP components were found to be modulated by the affective meaning of words: The early posterior negativity (EPN), a component that is larger for emotion-laden words compared to neutral ones (Kissler et al., 2007, 2009; Herbert et al., 2008; Schacht and Sommer, 2009; Conrad et al., 2011; Keuper et al., 2014), appears around 200–300 ms after stimulus onset. It was first reported in the context of emotional face and picture processing (Junghöfer et al., 2001; Schupp et al., 2003, 2004), hence presumably reflecting general, modality-independent affective processing. The EPN is assumed to mirror fast and effortless detection of emotionally significant stimuli and thereby indexes natural selective attention (Olofsson et al., 2008). MEG studies reported that the neural loci of cognitive functions such as semantic memory, attention, and evaluation of emotional stimuli are involved in the formation of the EPN (Keuper et al., 2014). Furthermore, the late positive complex (LPC), appearing around 400–700 ms after stimulus onset, also proved sensitive to differences in the affective meaning of words (Dillon et al., 2006; Kissler et al., 2009; Schacht and Sommer, 2009; Conrad et al., 2011). This late component is assumed to indicate more elaborated and task-dependent cognitive processing of affective or emotional stimuli. This includes, for example, continued stimulus evaluation such as categorization or memory updating. Useful reviews on ERP emotion effects in visual word recognition have been provided by Citron (2012) or Kotz and Paulmann (2011).

To investigate potential effects of affect encoded at the sublexical phonological level within the framework of known general emotion effects during visual word recognition, we used a design including a classical manipulation of *lexical affective content* together with a novel manipulation of *sublexical affective potential* in a standard visual lexical decision task.

Most theoretical reasoning on *phonological iconicity* assumes phonology as the source of respective effects. If these effects exist, they should, though, also show and might most effectively be studied during silent reading which has been shown to involve mandatory phonological processing (e.g., Van Orden, 1987; Abramson and Goldinger, 1997; Ziegler et al., 2001; Conrad et al., 2007; Braun et al., 2009). The visual lexical decision task is the most standardized and most used research tool in the field of psycholinguistics. German is a shallow orthography with high grapheme-to-phoneme consistency, i.e., the presentation of specific German letter strings would evoke unambiguous phonological activations regardless of context and of whether a letter string is a word or not. Using a standard visual lexical decision task appears thus a reasonable initial step for the investigation of phonological iconicity effects in German. It provides both a methodological match to the available literature on emotion effects quoted above as well as an optimally standardized experimental context excluding potential distortion through auditory effects of, e.g., affective prosody or speaker identity.

At both the lexical and the sublexical level, our manipulations of *affective content* or *potential* involve the contrast between high arousal in combination with negative valence on the one hand, and low arousal combined with neutral valence on the other hand. This has both pragmatic and theoretical reasons: As already evident from Võ et al. (2009) and Schmidtke et al. (2014b), valence and arousal values of German words are characterized by a very tight correlation within the range of overall negative valence, but not within the positive valence range. That is, increasingly negative valence of concepts is generally associated with increasing arousal, whereas positive concepts can be either calm or exciting. As the SAVs we use for the operationalization of the *sublexical affective potential* represent the average values of words containing a given phonological segment, it goes—to some extend—by itself that comparable correlations are given for SAVs. That is, the majority of phonological segments with negative valence also have rather high arousal levels, whereas positive valence and arousal SAVs are less related. Further, the combination of negative valence and high arousal fits best the assumed reason underlying these *phonological iconicity* phenomena: the encoding of threat at a sublexical level (see Conrad et al., in preparation). Most of the phonological segments that might in general serve as icons of affective content—displaying statistically significant deviations from global neutral means—in the database of German words indeed follow this pattern of combining negative valence with high arousal. That is why the combination of negative valence and high arousal contrasted against neutral valence and low arousal allows for a most pronounced contrast—potentially leading to most pronounced effects—for this novel manipulation of *sublexical affective potential* taking into account both dimensions of the affective space.

As already mentioned, when considering phonological segments of syllabic onsets, nuclei, and codas rather than single phonemes, affectively deviant segments of negative valence and high arousal often also are structurally more complex—i.e., contain more phonemes—and of lesser frequencies of occurrence as compared to affectively neutral ones. To account for both types of effects—intrinsic SAVs on the one hand and formal salience on the other—as two potentially additive sources of *phonological iconicity* influencing affective processing during language perception, we prepared two separate experimental stimulus sets to be presented in one and the same experimental session (see Conrad et al., 2007, 2009, for detailed elaboration of the methodological advantages of this approach):

– Set 1 involves the natural confound of SAVs with formal salience to capture a most natural picture of effects of affective *phonological iconicity* or *sublexical affective potential*—just the way they arise in the lexicon.
– Set 2 controls for this confound to allow for a clearer attribution of possible *sublexical affective potential* effects, disentangling them from phenomena of structural complexity or frequency of occurrence.

We predict effects of the sublexical manipulation to be strongest when SAVs are allowed to co-vary with formal salience. Further, if any effects at all would still be obtained for the sublexical manipulation controlling for formal salience, these effects might—with even more confidence—be considered evidence for sublexical encoding of affectivity, especially if they resembled ERP effects established so far for general emotion processing during lexical decision, and predicted for our second factor—affective content at the lexical level. In particular, such effects might be expected similar to an EPN, because sublexical effects should occur rather early during the time course of the reading process—or at least not later than lexical effects.

## Materials and methods

### Participants

Forty-one native speakers of German, university students of the Freie Universität Berlin, participated in the experiment after giving informed consent. All were right-handed (Oldfield, 1971) with normal or corrected-to-normal vision. None of them reported neurological or language problems. Six participants were excluded from the final data analysis due to bad signal-to-noise ratio of ERP data so that data from 35 subjects (21 women; age range: 18–36 years, *M* = 26.7 years, *SD* = 4.2) were submitted to analyses. All participants received financial compensation.

### Stimuli and design

We selected two separate sets (set1: *maximally manipulated*; set 2: *maximally controlled*) of 312 German words each—containing between one and three syllables, with a maximum of nine letters length—from the extended BAWL database (Võ et al., 2009; publication of the extended version in preparation) as stimuli for the two experimental sets. Both sets involved twofold, independent manipulations of these two factors (each factor cell comprised 156 stimulus words):

– *Lexical affective content* (negative valence and high arousal vs. neutral valence and low arousal)

and

– *Sublexical affective potential* (negative valence and high arousal vs. neutral valence and low arousal, based on mean SAVs per word)

*Lexical affective content* was closely controlled for between the two cells of *sublexical affective potential* and vice versa.

L*exical affective content* is operationalized in the database in form of rating values of valence on a scale from −3 to 3, and of arousal on a scale from 1 to 5. A word was entered in the *negative high-arousing lexical affective content* condition when the mean of its valence ratings in the database was more negative than −0.8 (furthermore, the sum of mean and standard deviation of the valence ratings for a word did not exceed 0) and its arousal ratings higher than 2.8. For the *neutral low-arousing lexical affective content* condition the valence ratings of the words had to be between −0.8 and 0.8 (and the standard deviation below 1) and the arousal ratings lower than 2.8.

The factor *sublexical affective potential* was operationalized as follows: We computed hypothetical affective values for sublexical segments (the aforementioned *sublexical affective values*—SAVs) as a function of the affective values of the words they occur in in our database of over 6000 German words (Conrad et al., in preparation): We calculated valence and arousal SAVs for all given syllabic onsets, nuclei, and codas by averaging the rating values of words they form part of. We then averaged these values for all segments found in a single given word to obtain an estimate of the *sublexical affective potential* of this word. Naturally, the resulting scale widths for valence (−0.7–0.7) and arousal (2.5–3.2) of these *sublexical affective potential* values per word were much narrower than those of the *lexical affective content* rating scales. A word was entered in the negative high-arousing *sublexical affective potential* condition when its valence value was more negative than -0.05, and its arousal value higher than 2.9. For the neutral low-arousing *sublexical affective potential* condition the valence value of a word had to be between −0.04 and 0.45, and the arousal value lower than 2.9. Specifically for the sublexically neutral low-arousing words, additional attention was paid to the following selection criteria: If words contained single very negative or high-arousing phonological segments—albeit the overall mean fit in the neutral low-arousing category—they were excluded, for we assume that such single salient phonological segments could already attract enough attention to not let the whole word sound affectively “neutral” anymore. Stimulus characteristics are shown in Table 1. While our manipulation of *sublexical affective potential* is based on numerical mean SAVs across all phonological segments in a word, this certainly involves that specific segments are more likely to occur in one condition, e.g., negative/high arousal *sublexical affective potential*, than in the other (neutral/low arousal). To make our manipulation more transparent to the reader, Table 2 lists how many times specific phonological segments were used across conditions.

**Table 1:** **Means and standard deviations for manipulated variables and some of the control variables plus ***p***-values for tests of significant mean differences between the respective two conditions**.

	**Lex. Val**.		**Lex. Aro**.		**Sublex. Val**.		**Sublex. Aro**.		**LogFreq** + **1**		**Letters**		**Syllables**		**Orth. neighbors**		**LogBigram Freq** + **1**		**CVC complexity**	
	***M***	***SD***	***M***	***SD***	***M***	***SD***	***M***	***SD***	***M***	***SD***	***M***	***SD***	***M***	***SD***	***M***	***SD***	***M***	***SD***	***M***	***SD***
**MAXIMALLY MANIPULATED STIMULUS SET**																				
**Lexical affective content**																				
Negative valence high arousal	−1.58	0.50	3.62	0.42	−0.08	0.10	2.90	0.06	2.18	0.73	6.08	1.35	1.91	0.69	1.89	2.16	3.42	0.39	1.39	0.52
Neutral valence low arousal	0.11	0.38	2.33	0.29	−0.08	0.10	2.90	0.06	2.22	0.86	6.04	1.30	1.92	0.65	2.19	2.50	3.42	0.42	1.33	0.51
*p*	0.00		0.00		0.85		0.72		0.69		0.80		0.93		0.28		0.88		0.38	
**Sublexical affective potential**																				
Negative valence high arousal	−0.72	0.96	3.00	0.75	−0.15	0.09	2.94	0.05	2.24	0.80	6.17	1.32	1.68	0.63	1.88	2.05	3.36	0.39	1.63	0.57
Neutral valence low arousal	−0.74	0.96	2.95	0.73	−0.01	0.03	2.85	0.03	2.17	0.80	5.96	1.33	2.15	0.63	2.21	2.59	3.48	0.41	1.09	0.24
*p*	0.86		0.53		0.00		0.00		0.47		0.15		0.00		0.22		0.01		0.00	
**MAXIMALLY CONTROLLED STIMULUS SET**																				
**Lexical affective content**																				
Negative valence high arousal	−1.60	0.46	3.59	0.40	−0.06	0.10	2.90	0.06	2.06	0.79	6.22	1.48	1.93	0.71	1.58	1.87	3.39	0.35	1.38	0.44
Neutral valence low arousal	0.11	0.38	2.32	0.28	−0.05	0.10	2.89	0.06	2.06	0.80	6.15	1.45	1.94	0.69	1.76	2.30	3.37	0.39	1.39	0.44
*p*	0.00		0.00		0.44		0.88		0.95		0.67		0.87		0.46		0.67		0.88	
**Sublexical affective potential**																				
Negative valence high arousal	−0.76	0.94	2.98	0.71	−0.12	0.07	2.94	0.04	2.01	0.77	6.28	1.43	1.92	0.70	1.61	1.96	3.37	0.39	1.39	0.45
Neutral valence low arousal	−0.74	0.97	2.94	0.74	0.02	0.08	2.85	0.05	2.11	0.81	6.08	1.49	1.95	0.71	1.73	2.23	3.39	0.34	1.38	0.42
*p*	0.89		0.68		0.00		0.00		0.28		0.23		0.75		0.60		0.66		0.72	

*Lex. Val., lexical valence ratings; Lex. Aro., lexical arousal ratings; Sublex. Val., computed sublexical valence values; Sublex. Aro., computed sublexical arousal values; LogFreq + 1, word frequency in terms of dec. logarithms + 1; Letters, number of letters; Syllables, number of syllables; Orth. Neighbors, number of orthographic neighbors; LogBigramFreq + 1, mean positional bigram frequencies in terms of dec. logarithms + 1; CVC complexity, combined consonant complexity patterns of each syllable*.

**Table 2:** **Phoneme (segments) distribution (in DISC Phonetic Encoding Convention; Burnage, 1990) across the conditions of ***sublexical affective potential*** in both stimuli sets**.

**Phonemes**	**Maximally controlled set**		**Maximally manipulated set**	
	**neg-high**	**neut-low**	**neg-high**	**neut-low**
**ONSETS**				
=	16	6	10	1
= v	1	0	3	0
b	6	18	5	25
bl	2	5	1	0
br	6	0	10	0
d	14	14	10	31
dr	5	0	8	0
f	16	7	9	7
fl	0	6	0	2
fr	0	13	0	1
g	11	19	2	30
gl	0	2	0	2
gn	1	1	0	2
gr	5	0	4	0
h	8	5	6	9
k	4	20	9	18
kl	0	9	0	3
kn	2	0	6	0
kr	11	0	17	0
ks	3	0	1	0
l	6	26	1	34
m	12	19	8	33
n	15	8	8	21
N	2	2	2	2
p	8	18	7	9
pr	4	1	5	0
r	33	6	25	5
s	7	0	8	0
S	14	0	13	0
Sl	1	0	3	0
Sp	0	0	2	0
Sr	1	0	4	0
st	2	0	3	0
St	4	11	3	1
Str	2	0	10	0
Sv	0	0	3	0
t	27	17	16	21
tr	4	0	12	0
v	7	16	5	19
x	3	4	4	5
z	6	17	3	31
**NUCLEI**				
&	37	35	30	22
)	3	3	1	3
/	1	0	2	0
@	61	79	71	106
|	6	0	3	0
a	8	21	6	29
B	5	5	3	4
e	11	18	2	18
E	38	18	35	12
i	18	21	10	25
I	43	16	29	17
o	13	13	7	29
O	7	22	15	15
u	2	12	0	10
U	15	13	19	9
W	12	15	13	27
X	13	1	8	1
y	2	3	3	3
Y	5	8	5	6
**CODAS**				
+	1	0	4	0
=	4	0	7	0
b	0	1	0	2
d	2	2	3	0
f	0	5	1	5
ft	1	0	1	1
g	8	0	1	0
k	10	12	15	4
l	6	26	6	35
ln	6	0	1	0
lt	2	4	2	0
lx	0	2	0	0
m	2	14	3	13
n	44	38	41	47
N	3	7	3	11
Nk	1	0	3	0
nt	0	22	0	5
p	12	0	11	0
r	36	29	26	31
r =	2	0	3	0
rk	0	2	0	2
rn	1	4	0	2
rS	2	0	2	0
rt	0	2	0	0
s	19	4	12	2
S	8	0	3	1
st	11	0	8	0
t	12	12	9	14
v	0	0	2	0
x	14	8	13	7
xt	6	0	7	0

*neg-high, combination of negative valence and high arousal; neut-low, combination of neutral valence and low arousal*.

In both sets a large number of variables that are known to influence visual word processing (see Graf et al., 2005, for an overview) were controlled for between cells of the two factors (see also Table 1):

– Word frequency (in terms of dec. logarithms + 1 of the word frequencies in the SUBTLEX database, Brysbaert et al., 2011)
– Word length in terms of number of letters (max = 9)/phonemes/syllables (max = 3)
– Imageability ratings
– Word class (nouns, verbs, adjectives)
– Stress pattern (on which syllable)
– Composita patterns (classification of prefixes, suffixes, composita of two words, loanwords)
– Number of orthographic and phonological neighbors (Coltheart et al., 1977)
– Frequency of orthographic and phonological neighbors (in terms of the dec. logarithm + 1 of the sum of the frequencies of all neighbors)
– Specifically the number of orthographic and phonological neighbors with higher frequencies

In the *maximally controlled set* we further controlled for the following sublexical variables:

– Syllable lengths (separately for each of the maximal three syllables and separately for orthographic and phonological syllables)
– Token frequency of the first syllable (dec. logarithms + 1; for first syllable frequency effects see Carreiras et al., 1993; Conrad and Jacobs, 2004; Hutzler et al., 2004)
– Token frequencies of all syllable segments (onset 1–coda 3, respectively, dec. logarithm + 1)
– Morphological (CVC) structure of the onsets, nuclei, and codas respectively in all syllables
– Combined consonant complexity patterns of each syllable (possible combinations: onset and coda simple [coded as 1], onset complex and coda simple [coded as 2], onset simple and coda complex [coded as 2], onset and coda complex [coded as 3])
– Lengths of the nuclei vowels in each syllable (short vs. long)
– Positional token frequencies (dec. logarithm + 1) of all bigrams and biphons in a word
– Token frequeny (dec. logarithm + 1) of the respective last bigram and biphon of a word
– Token frequency (dec. logarithm + 1) specifically of those bigrams spanning syllable boundaries

To assure best overall comparability between data for the two sets, all stimuli were presented in a unique experimental session to the same participants. Overlapping items, i.e., stimuli that were used in both manipulations, entered the final stimulus set only once to avoid repetition. Thus, a total set of 521 stimulus words was presented together with 535 pseudowords that were matched to word stimuli in length and number of syllables. Pseudowords included pseudohomophones to assure a sufficiently difficult overall task environment where participants actually had to achieve lexical access for stimulus words. The pseudoword material involved a different experimental manipulation not addressed in the present study. All results presented in this paper refer exclusively to the word material possessing affective values at both the lexical and (hypothetically) the sublexical level.

### Procedure

All Stimuli were presented visually in randomized order using “Times New Roman” font, size 24, in white letters on a black background in the center of a 17″ computer screen with 80 cm distance to the participant's eyes. Each trial began with the presentation of a fixation cross (500 ms) followed by a blank screen of 500 ms. The pseudo-randomized single word and pseudoword items were presented for 500 ms each and were followed by a blank screen that lasted until the key response had been carried out, followed by a scattered inter-stimulus interval of 700–1500 ms. The task of the participants was to decide whether the presented stimulus was a “word” or a “non-word” by pressing one of two respective push-buttons on a Playstation remote control. The labels “Wort” (word) and “Nichtwort” (non-word) were counterbalanced between left and right hand responses across participants. They were encouraged to respond as fast but also as accurately as possible. Before the actual experiment started, 10 initial practice trials (5 words, 5 pseudowords) were run. The whole experiment contained 1056 trials and was split into four blocks which lasted about 10–12 min each. In between these blocks participants were allowed to rest as long as they wished.

### EEG recording and (pre-)processing

The EEG was recorded from 61 AgCl-electrodes (Fp1, Fpz, Fp2, AF3, AF4, F5, F3, F1, Fz, F2, F4, F6, FT7, FC3, FC1, FCz, FC2, FC4, FT8, T7, C5, C3, C1, Cz, C2, C4, C6, T8, TP7, CP5, CP3, CP1, CPz, CP2, CP4, CP6, TP8, P9, P7, P5, P3, P1, Pz, P2, P4, P6, P8, P10, PO9, PO7, PO3, POz, PO4, PO8, PO10, O1, Oz, O2, Iz, M1, M2) fixed to the scalp via an elastic cap using two 32-channel amplifiers (BrainAmp, Brain Products, Germany). Electrodes were arranged according to the International 10–20 system (Jasper, 1958; American Electroencephalographic Society, 1991) and average impedances were kept below 2 kΩ. The electrooculogram (EOG) was monitored by two electrodes at the outer canthi of the participant's eyes and two electrodes above and below the right eye. EEG and EOG signals were recorded with a sampling rate of 500 Hz, referenced to the right mastoid, but re-referenced offline to linked mastoids. The AFz electrode was used as ground electrode. Later offline filtering included a bandpass filter of 0.1–20 Hz and a notch filter of 50 Hz. Independent component analysis (ICA; Makeig et al., 1996; Jung et al., 1998) was carried out to identify and remove eye movement artifacts. The continuous EEG signal was cut into segments of 950 ms total length, consisting of a 150 ms pre-stimulus baseline and an 800 ms post-stimulus interval. After baseline correction, trials containing artifacts were excluded from further analysis using an automatic artifact rejection: differences >80 μV in intervals of 70 ms or amplitudes >50 or <−50 μV were considered artifacts. Segments containing correctly answered word trials got averaged per condition, participant and electrode, before grand averages were computed across all participants. To visually compare the ERP signals of different conditions the (sublexically) neutral low-arousing words were always subtracted from the (sublexically) negative high-arousing words.

### Data analysis

#### Behavioral data

Mean correct response latencies and error rates of the word stimuli were submitted to separate ANOVAs—testing whether a potentially given effect generalizes over subjects (F1 analysis) and over items (F2 analysis)—for the factors *lexical affective content* (2) and *sublexical affective potential* (2).

#### EEG data

Time windows for the expected ERP components of the *lexical affective content* of words were defined based on the literature (see Citron, 2012) and visual inspection of the grand averages: 200–300 ms for the EPN, and 400–700 ms for the LPC.

For potential effects of the *sublexical affective potential* of the word stimuli, there are no prior studies to base hypotheses on. We thus used an exploratory approach where a time-line analysis with 20 ms time windows (starting from each data point) was carried out. To reduce the chances of false positives potentially arising through consecutive testing, only total time windows of at least 50 ms length—consisting of consecutively significant single time windows revealed by the time-line analysis—were used for further analysis (based on the approach suggested by Guthrie and Buchwald, 1991).

Repeated-measures ANOVAs were conducted with the mean activity [μV] values of the selected time windows using the software IBM SPSS Statistics. The ANOVAs involved the within-subject factors *lexical affective content* (2) or *sublexical affective potential* (2). In order to assess topographical potential distributions of relevant effects over the scalp through an *a priori* designed, hypothesis-independent approach using data from a maximum of electrodes, the ANOVAs further included the topographic factors left-mid-right (3) and anterior-central-posterior (3). For these topographic analyses the scalp electrodes were subdivided into the following 9 clusters of 6 electrodes each: right anterior (FP2, AF4, F4, F6, FC4, FT8), mid anterior (F1, Fz, F2, FC1, FC2, FCz), left anterior (FP1, AF3, F3, F5, FC3, FT7), right central (C4, C6, T8, CP4, CP6, TP8), mid central (C1, Cz, C2, CP1, CPz, CP2), left central (C3, C5, T7, CP3, CP5, TP7), right posterior (P4, P6, P8, PO4, PO8, O2), mid posterior (P1, Pz, P2, POz, Oz, Iz), and left posterior (P3, P5, P7, PO3, PO7, O1).

**Figure 1:**
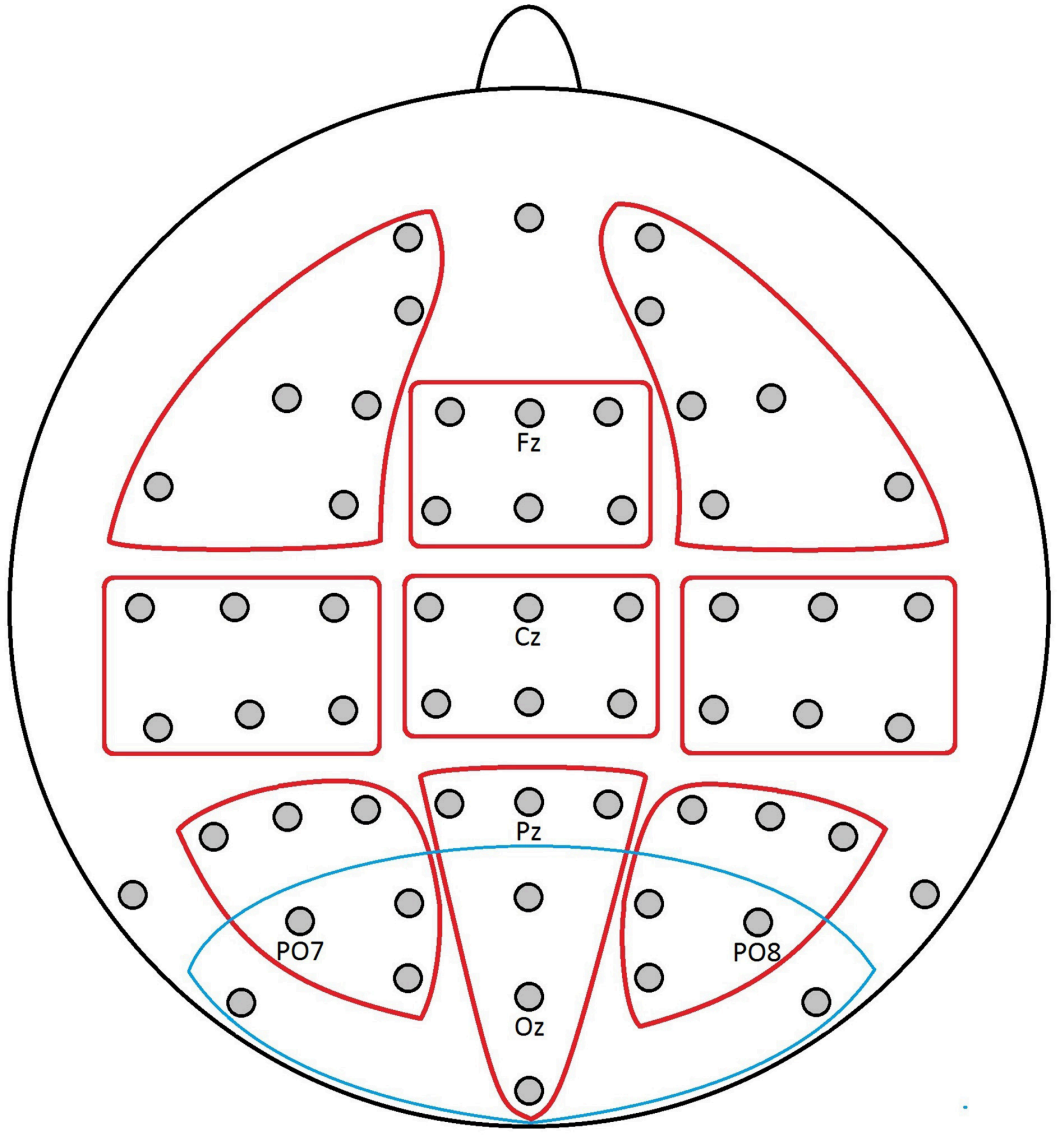
**Electrode positions of the applied 10–20 system with marked topographic clusters (ROIs) as used in the analyses: red, exploratory topographic clusters; blue, EPN ROI**.

Furthermore, a region of interest (ROI) for the EPN was defined using a cluster of the 11 most posterior electrodes (PO9, PO7, PO3, POz, PO10, PO8, PO4, O1, Oz, O2, Iz), based on earlier topographic data regarding EPN effects in our research group (Conrad et al., 2011; Recio et al., 2014). If the visual topography patterns suggested so, data of the EPN ROI were submitted to paired *t*-tests between the affective conditions. The combination of these two approaches toward topographic analysis, one unbiased and one guided by hypotheses, should offer a most comprehensive insight in this novel research topic. All topographic clusters and the ROI are displayed in Figure 1.

Greenhouse-Geisser corrected *p*-values (Greenhouse and Geisser, 1959) are reported for all ANOVA results. Significant interactions with topographic factors were followed up by paired *t*-tests within the respective topographic clusters. The *p*-values of multiple *post-hoc t*-tests got Bonferroni-Holm adjusted (Holm, 1979) and are marked as p_adj_. As measure of effect size $\eta_{\text{p}}^{2}$ is reported for the ANOVAs (Keppel, 1991; Tabachnick and Fidell, 2001) and Pearson's r for the *t*-tests (Clark-Carter, 2003; Field, 2009).

## Results

### Behavioral results

#### Maximally manipulated stimulus set

The analysis of reaction times (RTs) for the *sublexical affective potential* yielded no significant differences between the RTs to sublexically negative high-arousing words and to sublexically neutral low-arousing words [*F1*_(1, 40)_ = 3.66, *p* = 0.06, η_p_^2^ = 0.08; *F2*_(1, 306)_ = 1.45, *p* = 0.23, η_p_^2^ = 0.01]. For the *lexical affective content*, we found a significant F1 effect (with slower responses to negative high-arousing words than to neutral low-arousing words), but the F2 analysis remained non-significant [*F1*_(1, 40)_ = 6.35, *p* = 0.02, η_p_^2^ = 0.14; *F2*_(1, 306)_ = 1.01, *p* = 0.32, η_p_^2^ = 0.003]. Regarding error rates, again we do not find a significant effect for *sublexical affective potential* [*F1*_(1, 40)_ = 3.43, *p* = 0.07, η_p_^2^ = 0.08; *F2*_(1, 306)_ = 1.28, *p* = 0.26, η_p_^2^ = 0.004]. There is also no effect for error rates regarding the *lexical affective content* [*F1*_(1, 40)_ = 0.01, *p* = 0.91, η_p_^2^ = 0.00; *F2*_(1, 306)_ = 0.00, *p* = 0.99, η_p_^2^ = 0.00].

#### Maximally controlled stimulus set

Although, the F1 analysis of RTs renders a significant effect for the *sublexical affective potential* with faster responses to the sublexically neutral low-arousing words, the F2 analysis is non-significant [*F1*_(1, 40)_ = 5.56, *p* = 0.02, η_p_^2^ = 0.12; *F2*_(1, 305)_ = 1.29, *p* = 0.26, η_p_^2^ = 0.004]. Also for the *lexical affective content*, there is no significant effect in RTs' analysis [*F1*_(1, 40)_ = 2.96, *p* = 0.09, η_p_^2^ = 0.07; *F2*_(1, 305)_ = 1.93, *p* = 0.17, η_p_^2^ = 0.01]. Looking at the error rates, a significant F1 difference between lexical affective conditions (more errors on negative high-arousing words compared to neutral low-arousing ones) is not accompanied by a significant F2 analysis [*F1*_(1, 40)_ = 5.52, *p* = 0.02, η_p_^2^ = 0.12; *F2*_(1, 305)_ = 1.03, *p* = 0.31, η_p_^2^ = 0.003]. Further, there is no significant effect for the *sublexical affective potential* [*F1*_(1, 40)_ = 1.67, *p* = 0.2, η_p_^2^ = 0.04; *F2*_(1, 305)_ = 0.27, *p* = 0.6, η_p_^2^ = 0.001].

### ERP results

#### Maximally manipulated stimulus set

##### Lexical affective content

**Figure 2:**
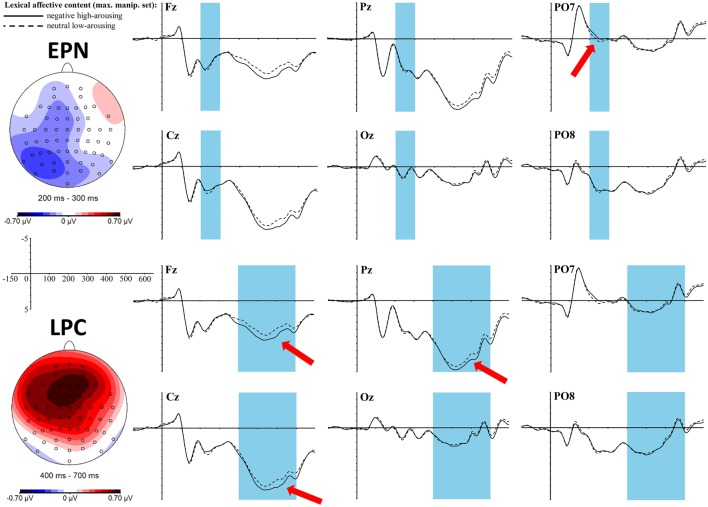
**ERP effects of the ***lexical affective content*** (top: EPN, bottom: LPC) in the ***maximally manipulated*** stimulus set at selected electrode sites**. The red arrows indicate at which electrodes the effects are most pronounced. For the topographic maps neutral low-arousing words were subtracted from negative high-arousing words.

An early effect of the *lexical affective content* was found in the time window of the EPN between 200 and 300 ms in interaction with the topographic factor left-mid-right [*F*_(2, 68)_ = 3.7, *p* = 0.03, η_p_^2^ = 0.1]. *T*-tests within each of the three laterality clusters only showed a trend toward a difference between neutral low-arousing and negative high-arousing words in the left cluster [*t*_(34)_ = −2.17, *p*_adj_ = 0.12, *r* = 0.35] with a larger negativity for negative high-arousing words. Yet, the topographic map (Figure 2) reveals that this negativity is of a shape that cannot be caught well by the cluster formation of the exploratory topographic analysis. Rather, most distinct negativity shows in a left posterior area, as would be hypothesized for the expected EPN. Results of EPN ROI analysis were: *t*_(34)_ = −1.87, *p* = 0.07, *r* = 0.31. Although, here again, we can only find a trend toward significance, in both analyses the postulated effect is of a medium size, which cannot be neglected (see discussion for why the effect might not be as strong as in previous literature).

A late positive complex (LPC) can be found between 400 and 700 ms as a significant main effect for the *lexical affective content* [*F*_(1, 34)_ = 8, *p* = 0.01, η_p_^2^ = 0.19] with more positive values for the negative high-arousing words compared to the neutral low-arousing words. Furthermore, we find a significant interaction of this lexical effect with the topographic cluster division anterior-central-posterior [*F*_(2, 68)_ = 7.88, *p* = 0.004, η_p_^2^ = 0.19]: *t*- tests within each of these clusters revealed significant differences between the two *lexical affective content* conditions in the anterior [*t*_(34)_ = 4.12, *p*_adj_ < 0.003, *r* = 0.58] and the central cluster [*t*_(34)_ = 2.74, *p*_adj_ = 0.02, *r* = 0.43]. This fronto-central positivity is also reflected in the topographic map as shown in Figure 2.

##### Sublexical affective potential

**Figure 3:**
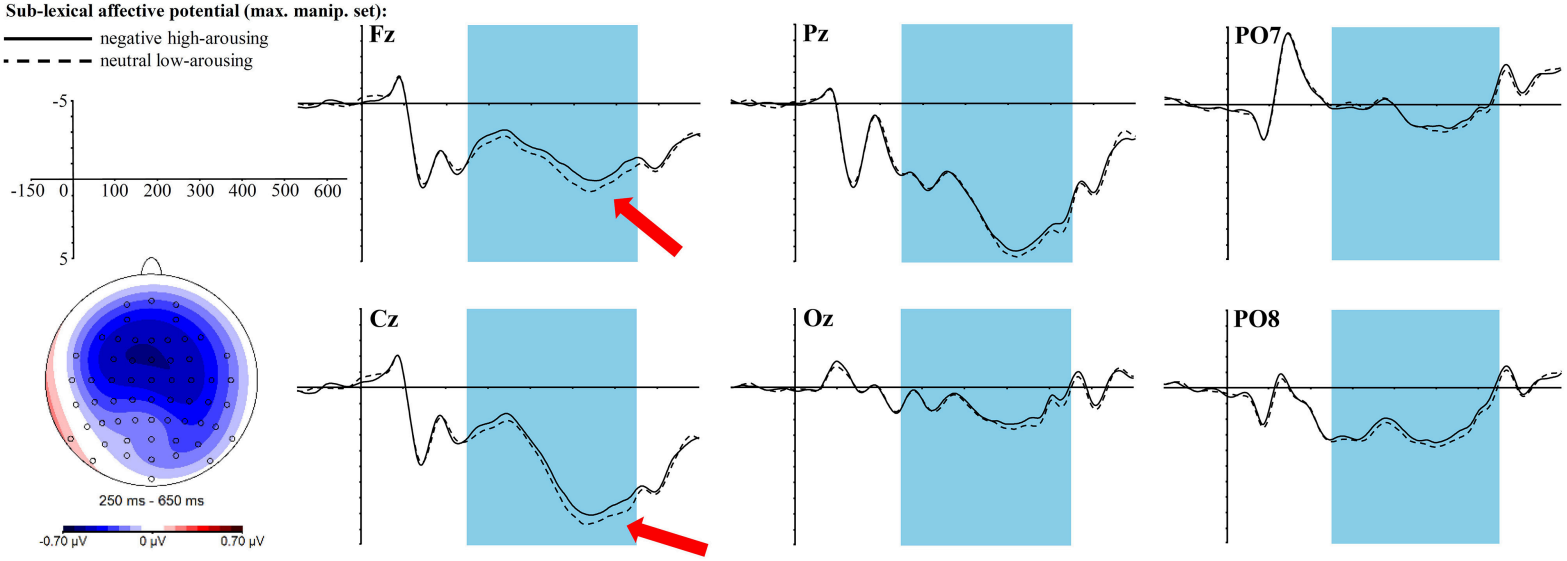
**ERP effect of the ***sublexical affective potential*** in the ***maximally manipulated*** stimulus set at selected electrode sites**. The red arrows indicate at which electrodes the effect is most pronounced. For the topographic map sublexically neutral low-arousing words were subtracted from sublexically negative high-arousing words.

Visual inspection already suggested a robust and long-lasting negativity between 250 and 650 ms that proved to be a significant main effect of the *sublexical affective potential* [*F*_(1, 34)_ = 7.77, *p* = 0.01, η_p_^2^ = 0.19] with sublexically negative high-arousing words eliciting a larger negativity over this whole time interval than sublexically neutral low-arousing words. Also the 3-fold interaction of *sublexical affective potential* × topographic factor anterior-central-posterior × topographic factor left-mid-right turns out significant [*F*_(4, 136)_ = 4.76, *p* = 0.003, η_p_^2^ = 0.12]. After correction for multiple testing, one of the *t*-tests in each of the nine topographic clusters turned out significant [in the right central cluster with *t*_(34)_ = −3.06, *p*_adj_ = 0.036, *r* = 0.46], one marginally significant [in the right posterior cluster with *t*_(34)_ = −2.86, *p*_adj_ = 0.056, *r* = 0.44], and four more neighboring clusters still showed trends [left anterior cluster with *t*_(34)_ = −2.42, *p*_adj_ = 0.11, *r* = 0.38, mid anterior cluster with *t*_(34)_ = −2.57, *p*_adj_ = 0.09, *r* = 0.4, right anterior cluster with *t*_(34)_ = −2.32, *p*_adj_ = 0.11, *r* = 0.37, and mid central cluster with *t*_(34)_ = −2.74, *p*_adj_ = 0.07, *r* = 0.43], always with a larger negativity for sublexically negative high-arousing words. Figure 3 displays the topography of this right-central negativity and the ERP graphs at selected electrodes.

#### Maximally controlled stimulus set

##### Lexical affective content

**Figure 4:**
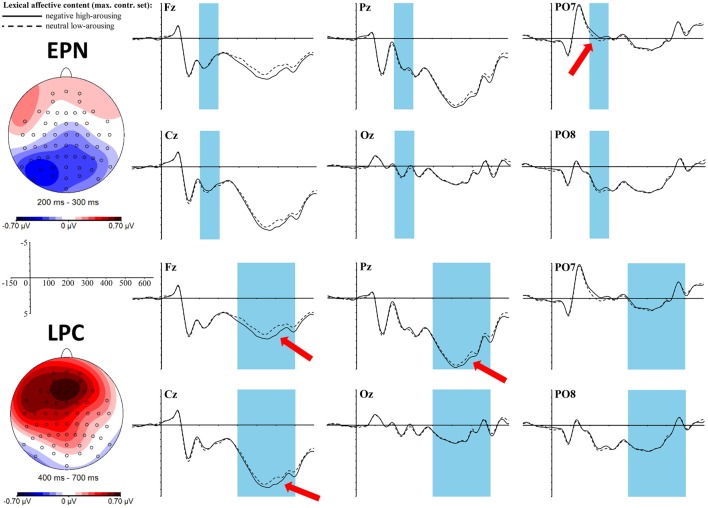
**ERP effects of the ***lexical affective content*** (top: EPN, bottom: LPC) in the ***maximally controlled*** stimulus set at selected electrode sites**. The red arrows indicate at which electrodes the effects are most pronounced. For the topographic maps neutral low-arousing words were subtracted from negative high-arousing words.

In the EPN time window between 200 and 300 ms an early effect of *lexical affective content* exists in interaction with the topographic factor anterior-central-posterior [*F*_(2, 68)_ = 8.23, *p* = 0.003, η_p_^2^ = 0.2] as well as in a 3-fold interaction also including the left-mid-right factor [*F*_(4, 136)_ = 3.68, *p* = 0.01, η_p_^2^ = 0.1]. *T*-tests within the respective topographic clusters reveal a significant difference between neutral low-arousing and negative high-arousing words in the whole posterior cluster [*t*_(34)_ = −2.71, *p*_adj_ = 0.03, *r* = 0.42] as well as trends in the single posterior clusters: left posterior [*t*_(34)_ = −2.88, *p*_adj_ = 0.06, *r* = 0.44], mid posterior [*t*_(34)_ = −2.46, *p*_adj_ = 0.13, *r* = 0.39], and right posterior [*t*_(34)_ = −2.5, *p*_adj_ = 0.14, *r* = 0.39], always showing a higher negativity for the lexically negative and high-arousing words. A *t*-test within the EPN ROI shows a significant difference between the two lexical affective conditions [*t*_(34)_ = −3.17, *p* = 0.003, *r* = 0.48] going in the same direction. The topography of this effect does well reflect the EPN pattern as expected. It is shown together with the EEG graphs at selected electrodes in Figure 4 (upper part).

A late positive complex (LPC) shows between 400 and 700 ms as a significant main effect for *lexical affective content* [*F*_(1, 34)_ = 6.16, *p* = 0.02, η_p_^2^ = 0.15] with more positive values for the negative high-arousing words compared to neutral low-arousing words. Also the interaction of *lexical affective content* with the topographic division anterior-central-posterior is significant [*F*_(2, 68)_ = 8.04, *p* = 0.01, η_p_^2^ = 0.19], with a significant *t*-test result in the anterior cluster [*t*_(34)_ = 3.71, *p*_adj_ = 0.003, *r* = 0.54] as well as a trend showing within the central cluster [*t*_(34)_ = 2.22, *p*_adj_ = 0.07, *r* = 0.36]. This fronto-central positivity with negative high-arousing words displaying a higher positivity than neutral low-arousing words is displayed in the lower topographic map of Figure 4.

##### Sublexical affective potential

**Figure 5:**
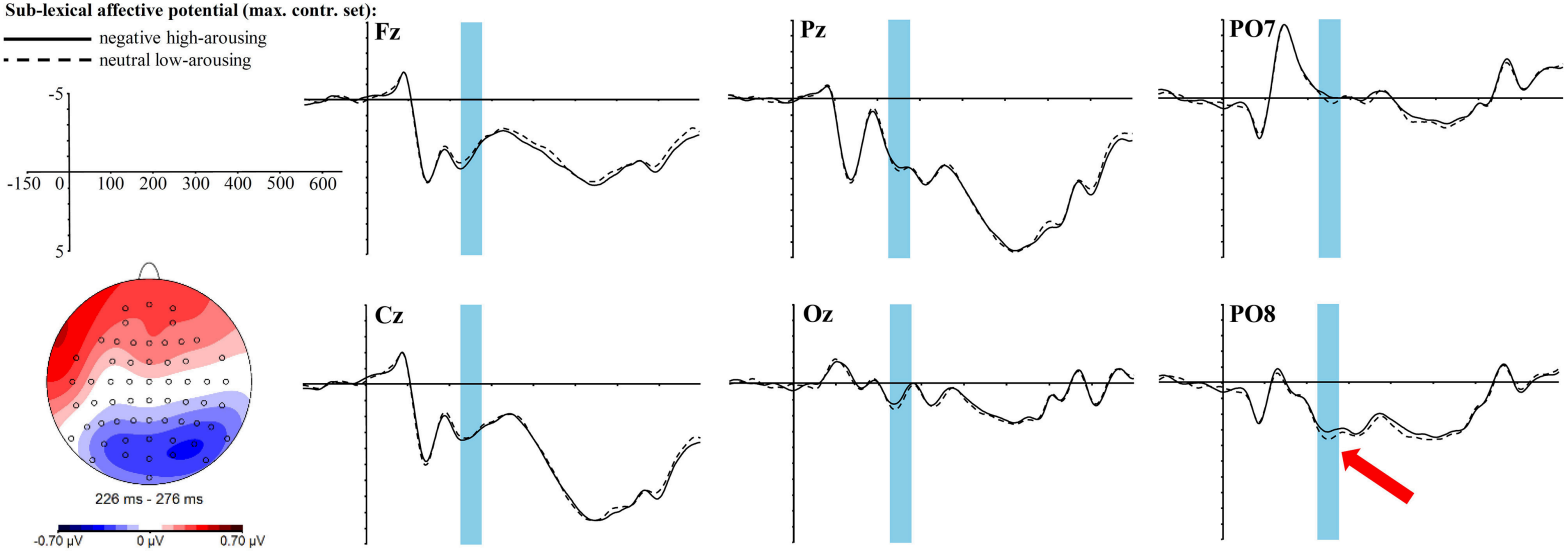
**ERP effect of the ***sublexical affective potential*** in the ***maximally controlled*** stimulus set at selected electrode sites**. The red arrow indicates at which electrode the effect is most pronounced. For the topographic map sublexically neutral low-arousing words were subtracted from sublexically negative high-arousing words.

The exploratory time-line analysis revealed contiguous significant time windows between 226 and 276 ms for the interaction of the *sublexical affective potential* with the topographic factors anterior-central-posterior. Thus, we analyzed this time window as a whole, which yields a significant interaction of *sublexical affective potential* with the anterior-central-posterior clustering [*F*_(2, 68)_ = 6.67, *p* = 0.01, η_p_^2^ = 0.16]. Solving this interaction only leads to a rough trend within the whole posterior cluster [*t*_(34)_ = −1.9, *p*_adj_ = 0.2, *r* = 0.31] with a more negative amplitude for the sublexically negative high-arousing words, yet of medium effect size. Visual inspection of the topographic map (see Figure 5) reveals that this posterior negativity looks quite similar to the lexical EPN. Hence, we also tested for significance within the EPN ROI: the *t*-test shows a significant difference between sublexically negative high-arousing words and sublexically neutral low-arousing words [*t*_(34)_ = −2.68, *p* = 0.01, *r* = 0.42]. The topography and ERP graphs at selected electrodes are displayed in Figure 5.

## Discussion

The present study investigates whether systematic sound-to-meaning correspondences that we had detected in the German language influence the neural processes of language perception—assessed by EEG recordings during the most standard task used in psycholinguistic research: visual lexical decision.

There is a longstanding debate in theoretical linguistics oscillating between the well-known axiom of arbitrary relations between the signifier and the signified on the one hand, and numerous studies on phenomena of *sound symbolism* and *phonological iconicity* on the other hand (for reviews see Perniss et al., 2010; Schmidtke et al., 2014a; Dingemanse et al., 2015).

Here, we focused on sound-to-meaning correspondences assumed to represent *phonological iconicity* with regard to a sublexical encoding of affect: Certain phonological segments—syllabic onsets, nuclei or codas—were found to occur particularly often in words of negative and/or high-arousing semantic meaning. As these findings proved statistically reliable across a large-scale database of over 6000 German words, we assume they might represent a certain degree of iconic organization of language rather than merely idiosyncratic “Gestalt” features of single words (Conrad et al., in preparation).

Based on this assumption, we calculated:

– First, *sublexical affective values* (SAVs) for single phonological segments as a function (average) of the affective values of all words they occur in
– Second, an estimate of the *sublexical affective potential* of whole words as a function (average) of the SAVs of all phonological segments forming this word

We then tested—using EEG measurements—whether apparent sound-to-meaning correspondences represent anything more than a hard-to-interpret “intriguing finding” arising from statistical analyses of large-scale lexical databases. We used these measures of *sublexical affective potential—*derived directly from the large-scale database—as an experimental factor distinguishing between words that “should” sound—according to these sound-to-meaning correspondences in the database—highly arousing and negative vs. words with rather neutral phonological affective qualities.

Our data suggest that these sound-to-meaning correspondences or statistical regularities of German with regard to sublexical phonology and affective content of words are rooted in phenomena that crucially influence basic online reading processes: Regardless of the actual *lexical affective content* of stimuli, words that were composed of phonological segments typically occurring in words of negative high-arousing meaning caused a very robust and long-lasting negativity in the ERP signal when participants simply tried to lexically access these words—compared to words consisting of affectively “neutral” phonological segments. As the most important finding of our study, this effect is strong evidence for the psychological relevance of affective sound-to-meaning correspondences in the German language at the level of sublexical units.

However, it is more difficult to attribute this effect to a specific type of processing. This is because those phonological segments typically occurring in words with threatening affective content (high arousal and negative valence) tend to be of formal salience as well: their frequency of occurrence is considerably low and/or they are phonologically rather complex, i.e., combining several consonants in syllabic onsets or codas. Note that this makes perfectly sense from an evolutionary perspective: If language would choose a specific phonological segment as a sublexical sign of threatening affective content, it should use this sign not too often to avoid inflation or decay of the alerting sign character. Further, the alerting character of the sign would clearly benefit from salient perceptive characteristics such as, for instance, complex phonological structure requiring increasing effort for articulation processes for several consonants combined in one syllabic onset or coda. In a strict sense, this confound with structural saliency makes it difficult to interpret our robust effect for the manipulation of *sublexical affective potential* in the maximally manipulated set as anything else than an effect of general sublexical encoding processes during silent reading—arising from the complexity and/or low frequency of the sublexical units (see Nuerk et al., 2000, for phonological/subsyllabic component frequency; Goslin et al., 2006, for syllabic structure; Barber et al., 2004; Hutzler et al., 2004, for syllable frequency; Hauk et al., 2006a,b, for bigram frequency). According to a two-fold representation of phonological units comprising an auditory as well as motor template (Hickok, 2012), also articulatory activations—especially with regard to the complex phonological clusters—are possibly involved. Neuroimaging studies, indeed, show motor circuits responsible for articulatory movements to be activated in response to visually presented word stimuli (Hagoort et al., 1999; Burton et al., 2005).

To control for the influence of these potential intervenient factors we had prepared and presented an additional, *maximally controlled* stimulus set involving the same manipulations but controlling for the confounds of *sublexical affective potential* with formal complexity and frequency. In this set—though massively deteriorating the natural variance of the manipulated variable and respectively the strength of the manipulation—the *sublexical affective potential* of stimulus words still produced a small but significant effect in the ERP signal of non-neglectable medium effect size. More interestingly, the distribution of this effect across the scalp and the moment it appears during the reading process closely resemble what is typically reported—and also present in our data—for manipulations of affective content at the lexical level: an increased negativity at posterior electrode sites arising at around 200 ms after stimulus onset (EPN). Yet, although this topographic and temporal coincidence with the lexically driven EPN appears somewhat striking, this novel finding—obtained through explorative time-line analysis—certainly calls for corroboration in future studies that should also explore which brain regions may be involved in these processes.

Note also that both EPN and LPC effects of *lexical affective content* manipulations appear somewhat diminished in our data when compared to previous experimental reports focusing on general emotion effects during visual word recognition (e.g., Conrad et al., 2011; Recio et al., 2014; just to quote two from the same lab). In our study, these manipulations of *lexical affective content* only served as control measures allowing us to relate both the moment when effects of the *sublexical affective potential* would arise and how their morphology would look like in comparison to more classical effects of *lexical affective content* within one and the same experimental context. Such simultaneous manipulations of different factors that have to be kept independent from each other clearly have the consequence that the strength for each manipulation gets attenuated as compared to when manipulated alone. In consequence, resulting empirical effects may have got attenuated too.

Further, our specific manipulations of affective content combining negative valence with high arousal may not have favored lexical affective effects to show up in most robust ways, as these effects have been shown to be stronger for positive as compared to negative valence (Recio et al., 2014). We assume that this restriction to negative affective content may be responsible for the lack of effects in our behavioral data. Whereas a processing advantage for positive stimuli is consistently being reported in the literature, the picture is more heterogeneous for negative contents: One the one hand, the automatic evaluation hypothesis predicts faster processing of positive or negative words compared to neutral words, supported by several lexical decision studies (Hofmann et al., 2009; Kousta et al., 2009). However, also opposite findings, where reaction times for negative words are not different from neutral words (Briesemeister et al., 2012; Recio et al., 2014) or even longer compared to neutral or positive words (Carretié et al., 2008; Estes and Adelman, 2008) have been reported. Such findings are explained by the automatic vigilance hypothesis (Pratto and John, 1991), according to which fast and automatic evaluation of especially negative stimuli directs attention away from the actual task, e.g., lexical decision, causing prolonged response times and higher error rates due to a deeper processing of the negative word content or even because of a tendency to withdraw from negative stimuli.

The same may, of course, explain the absence of *sublexical affective potential* effects in our behavioral data. But note also that even though our ERP data show that this *sublexical affective potential* together with its formal salience do play a role for automatic reading processes, we do not see why this should necessarily bias—speed or delay—the tendency to decide that a given stimulus is a word or not. We do clearly not posit that these phenomena should—besides potentially attracting attention at some point of the reading process—trigger a fundamental general cognitive bias, and sublexical and lexical affective content are, further, unrelated in our stimuli. Taken together, the contrast between significant ERP effects and the lack of such effects at the behavioral level in our study may best serve as a good example of how RT effects only represent the final point of a decision process, whereas ERPs may better reveal fine-grained and potentially contradicting processes that precede a final response—concerning the latency of which their contradictory effects may have canceled each other out.

Whereas the topographical potential distribution of our early ERP effects aligns well with homogenous reports on classical EPN effects, the topography of the LPC effects deserves a bit more discussion, as in some studies, the LPC has been found to be more posterior (Herbert et al., 2008; Kissler et al., 2009). Yet in general, the amplitude, latency, and topographic dispersion of the LPC have been found to be task-dependent (Fischler and Bradley, 2006; Schacht and Sommer, 2009). Whereas a word counting task yielded a posterior LPC (Kissler et al., 2009), it showed a bit further central when subjects just had to passively listen to words (Herbert et al., 2008). With lexical decision tasks, the LPC usually is found in a fronto-central position (Schacht and Sommer, 2009; Conrad et al., 2011; Recio et al., 2014), and even further frontal when asking the participants to rate the words on affective dimensions (Dillon et al., 2006)—all latter reports being compatible to our findings *for lexical affective content*. On the other hand, we found no such typical LPC-like component for the contrast of *sublexical affective potential*. The reason therefore is probably that this component generally appears linked to higher-cognitive elaborative processing, whereas our sublexical manipulation taps into more basic processing stages.

What our data—obtained with highly controlled experimental manipulations and providing an excellent signal-to-noise ratio involving more than 150 stimuli per condition and 35 participants—suggest is that already specific phonological segments can trigger at the sublexical level what is classically observed and reported as (lexical) emotion effects during the reading process: an EPN at around 200 ms after stimulus onset. In combination with the finding of the long-lasting negativity in the less controlled stimulus set, our data thus represent novel neurophysiological evidence for *phonological iconicity* as a principle systematically influencing the organization of the vocabulary AND the online processing of a language like German. The reading system appears to be sensitive to the transport of affective information via sublexical signs of affective meaning. The EPN is usually interpreted as evidence for an early automatic attention shift toward emotionally relevant stimuli. So far, this emotional relevance was determined by the *lexical affective meaning* (or *content*) of word stimuli in a number of previous ERP studies (see Citron, 2012, for a review). In the case of our study, the same effect might already be elicited by sublexical phonological segments alone. One possibility of how this effect might arise can be seen in statistical learning: the sound-to-meaning correspondences our experimental manipulations are based upon could represent such well learned regularities, that presentation of certain phonological segments is sufficient to elicit the same emotional attention processes as whole word forms representing emotion-laden concepts. Phonological segments, in that case, would have acquired symbolic affective values via associative links across the lexicon. However, an alternative explanation would refer more directly to an internal relation between acoustic or phonological properties of specific phonological segments and affective meaning at the conceptual level: As we outline in Conrad et al. (in preparation), phonemes occurring more frequently in words of high arousal (and negative valence) tend to possess phonemic features—e.g., sibilants or unvoiced stops—that go along with an increasing arousal at the level of acoustic impressions, according to the *distinct features theory* by Jakobson et al. (1952). Therefore, it might have been the increasing arousal at the level of phonemic features typically occurring in words of high arousal and negative valence that has triggered the EPN in our data. This interpretation aligns with the general assumption of *phonological iconicity* to represent an internal relation between the conceptual and the sublexical level: Certain phonological segments—iconic for high arousal—could provoke the same pattern of electrophysiological activity—reflected by the EPN—as emotion-laden words, because the phonemic features of these segments are of similar affective salience. The fact that respective ERP effects of the *sublexical affective potential* appear as clearly diminished in the *maximally controlled* stimulus set compared to the *maximally manipulated* stimulus set is probably mainly due to the constraint of controlling for the major co-variation of *sublexical affective potential* with formal salience. But it has to be kept in mind that already this empirical confound *per se* sheds light on the *phonological iconicity* effects, as the German language apparently made use of phonological segments that leave most impressive “footmarks” in neural correlates of the language processing—as evident from the robust effects of our *maximal manipulation* of *sublexical affective potential*—to encode threatening affective meaning. Taken together, this pattern of findings strongly points toward an internal relation between sublexical signs and affective meaning at the conceptual level and is in clear opposition to the arbitrariness axiom of linguistic theory concerning the relation between a signifier and the signified.

Finally, note that also processes of production or articulation preparation may have influenced our ERP data for *sublexical affective potential*—even though the task was visual lexical decision. Phonological iconicity may well be rooted in articulation processes determining an internal relation between the conceptual and the sublexical level. This appears even more plausible considering the relation between SAVs and structural complexity of consonant syllabic segments (increasing complexity of negative/high arousal segments). As the motor theory of speech perception (Liberman and Mattingly, 1985) states, perception, and articulation aspects are highly entangled during neural processing of language (Pulvermüller et al., 2006; D'Ausilio et al., 2009), and our design does not allow to clearly distinguish between either perception or articulation preparation as potential sources of effects—which, in turn, appears a most fruitful field for future research.

Language comparisons could provide interesting insights concerning potentially “universal” vs. language-dependent features of *phonological iconicity*. In particular, as our data involve “phonological” iconicity effects after visual presentation using orthographic codes from a shallow orthography, it might be interesting to see whether similar effects could be obtained in languages with less transparent orthographies, e.g., using English words. If effects persisted for both consistent and inconsistent grapheme-to-phoneme mappings, this would suggest that iconicity with regard to affective content might have already generalized from the phonological to the orthographic domain.

## Author contributions

MC developed as principal investigator the idea for the project, got the funding, and was crucially involved—providing major contributions to all aspects of the work from stimulus selection, data analyses, to writing of the manuscript. SU conducted the experiment and analyzed the data, helped with stimulus preparation, and also wrote major parts of the manuscript. SK was involved in developing the idea, fundraising, interpreting the data, and preparation of the manuscript. DS was mainly involved in the corpus analyses behind this study and the calculation of the new SAVs. AA assisted through all steps of the experiment with his critical thinking and important feedback. AA and DS also contributed to conducting the EEG experiment. All authors contributed substantially to the conception of the experiment and the interpretation of the data, revised and approved the final manuscript and agreed to be accountable for all aspects of the work.

### Conflict of interest statement

The authors declare that the research was conducted in the absence of any commercial or financial relationships that could be construed as a potential conflict of interest.
